# Associations of hospitalisation – admission, readmission and length to stay – with multimorbidity patterns by age and sex in adults and older adults: the ELSI-Brazil study

**DOI:** 10.1186/s12877-023-04167-8

**Published:** 2023-08-21

**Authors:** Luciana Pereira Rodrigues, Diego Galdino França, João Ricardo Nickenig Vissoci, Nayara Malheiros Caruzzo, Sandro Rodrigues Batista, Cesar de Oliveira, Bruno Pereira Nunes, Erika Aparecida Silveira

**Affiliations:** 1 Graduate Program in Health Sciences, Faculty of Medicine, Federal University of Goiás, Goiânia, Brazil; 2 State University of Maringá, Maringá, Brazil; 3 Duke Global Health Institute, Duke University, Durham, NC USA; 4 Faculty of Medicine, Federal University of Goiás, Goiânia, Brazil; 5 Department of Health, Federal District Government, Brasília, Brazil; 6 Department of Epidemiology and Public Health, University College London, 1-19 Torrington Place, London, WC1E 6BT UK; 7 Faculty of Nursing, Federal University of Pelotas, Pelotas, Brazil

**Keywords:** Multimorbidity, Health care utilisation, ELSI-Brazil, Sex, Age groups, Network analysis

## Abstract

**Background:**

Although the association between multimorbidity (MM) and hospitalisation is known, the different effects of MM patterns by age and sex in this outcome needs to be elucidated. Our study aimed to analyse the association of hospitalisations’ variables (occurrence, readmission, length of stay) and patterns of multimorbidity (MM) according to sex and age.

**Methods:**

Data from 8.807 participants aged ≥ 50 years sourced from the baseline of the Brazilian Longitudinal Study of Ageing (ELSI-Brazil) were analysed. Multimorbidity was defined as ≥ 2 (MM2) and ≥ 3 (MM3) chronic conditions. Poisson regression was used to verify the association between the independent variables and hospitalisation according to sex and age group. Multiple linear regression models were constructed for the outcomes of readmission and length of stay. Ising models were used to estimate the networks of diseases and MM patterns.

**Results:**

Regarding the risk of hospitalisation among those with MM2, we observed a positive association with male sex, age ≥ 75 years and women aged ≥ 75 years. For MM3, there was a positive association with hospitalisation among males. For the outcomes hospital readmission and length of stay, we observed a positive association with male sex and women aged ≥ 75 years. Network analysis identified two groups that are more strongly associated with occurrence of hospitalisation: the cardiovascular–cancer–glaucoma–cataract group stratified by sex and the neurodegenerative diseases–renal failure–haemorrhagic stroke group stratified by age group.

**Conclusion:**

We conclude that the association between hospitalisation, readmission, length of stay, and MM changes when sex and age group are considered. Differences were identified in the MM patterns associated with hospitalisation according to sex and age group.

**Supplementary Information:**

The online version contains supplementary material available at 10.1186/s12877-023-04167-8.

## Introduction

The occurrence of hospitalisation varies by country and is associated with age, sex, and the characteristics of the local healthcare system, among others [1]. In the United States, in 2018, the percentage of people who had been hospitalised once or more was 9.3% in the age group 55 to 64 years and 23.3% among those aged 85 years or older, with no significant differences between sex [2], whereas in Brazil, in 2019, 37.4% of hospitalisations in the public health system occurred in the ≥ 50 age group, representing an expenditure of approximately 8 billion reais [3]. A recent systematic review evaluated the factors associated with hospitalisation, readmission, and length of stay and observed that multimorbidity or the co-occurrence of multiple chronic conditions in one individual increased the risk of these outcomes in the older adult population [4].

Multimorbidity may have different impacts on hospitalisation occurrence, readmission, and length of stay according to age and sex [5–9], but these impacts are not yet well elucidated in scientific literature. Some studies found an association between multimorbidity and hospitalisation in middle-aged adults, i.e., 45 to 64 years old, compared with older adults [5, 6]. However, others found a positive association in the older adult population [48, 8–10]. Regarding sex, there is evidence of higher hospitalisation prevalence associated with multimorbidity among males [6, 7]. Further research on the subject is needed to elucidate the different impacts of age and sex on the association between multimorbidity and hospitalisation outcomes.

Most studies that analysed the association between multimorbidity and hospitalisation used the simple disease count method [4, 10–12]. However, recent studies have suggested that identifying patterns of morbidities is a better way to measure multimorbidity and its outcomes in order to contribute to the improvement of actions and health care aimed at this population [13, 14]. Among the studies that analysed the association of the multimorbidity pattern with hospitalisation outcomes, readmission, and length of stay, the cardiovascular and metabolic morbidities groups were associated with these outcomes in most studies [15–19]. Moreover, to the best of our knowledge, only one study has investigated whether this association differs by sex, but only in the 85 years and older population [19]. Thus, further studies are needed to verify whether the multimorbidity pattern associated with hospitalisation, readmission, and length of stay changes according to sex and age, and to identify the possible specificities of each sex and age group to improve health care for these individuals.

In addition to regression models, which are commonly used in research, network analysis allows for the visualisation of complex interactions between network variables, thus contributing to understanding the studied phenomenon [20]. This type of analysis has already been used in some studies on multimorbidity and its associated factors [21, 22]. In this study, network analysis facilitates better visualisation and interpretation of the demographic variables sex and age concerning the association between hospitalisation and multimorbidity.

Hospitalisation in older adults can negatively affect their health, such as decreased functional capacity and quality of life, frailty, and hospital readmissions [23, 24]. Thus, identifying the morbidity patterns associated with this outcome is relevant and will contribute to direct prevention and health care actions. Our study aimed to analyse the association of hospitalisations’ variables (occurrence, readmission, length of stay) and patterns of multimorbidity (MM) according to sex and age among Brazilians aged ≥ 50 years.

## Methodology

### Sample and data

The present study is a cross-sectional study that utilised data from the baseline (2015–2016) of the Brazilian Longitudinal Study of Ageing (ELSI-Brazil), which relied on a representative sample of the Brazilian non-institutionalised population aged 50 years or older. This study’s data were collected via questionnaires administered at home by a trained team. To delimit the sample, the conglomerate sampling method was used, considering different stages of selection (municipalities, census sectors, and households) and estimating a sample of 10.000 individuals. The baseline of the study was composed of 9.412 residents from 70 municipalities in the five Brazilian regions. Informed consent was obtained from each participant of the study. The ELSI-Brazil was approved by the Research Ethics Committee of the Instituto René Rachou of the Oswaldo Cruz Foundation (protocol n. 34649814.3.0000.5091). All participants signed an informed consent form before the interviews. All regulatory and legal aspects were fulfilled.

### Variables

The following outcome variables were considered: (1) occurrence of hospitalisation, a dichotomous variable (yes or no) evaluated using the question ‘In the last 12 months, were you hospitalised for 24 hours or more?’; (2) readmissions, indicating the number of times the individual was hospitalised in the last 12 months; and (3) length of stay, indicating the duration of the most recent hospitalisation.

The following independent variables were included in this study: sex (female, male), age group (50 to 59 years, 60 to 74 years, 75 years or older), and multimorbidity. Multimorbidity was assessed from a list of 19 morbidities and the following question was asked for each morbidity:‘Has a doctor ever told you that you have…?’ with the response options: no, yes, and don’t know/no answer. The morbidities evaluated were hypertension, back problems, high cholesterol, cataract, arthritis or rheumatism, depression, diabetes, osteoporosis, heart problems (junction of the heart attack, angina, and heart failure morbidities), glaucoma, chronic obstructive pulmonary disease (COPD), haemorrhagic stroke, cancer, asthma, chronic renal failure, diabetic retinopathy, macular degeneration, Parkinson’s disease, and Alzheimer’s disease. Multimorbidity was defined as 2 morbidities and ≥ 3 morbidities [25]. For network analysis, morbidities were investigated separately, with binary values for each variable (i.e. 0 = no, 1 = yes).

### Statistical analysis

Data analysis was performed using R version 4.0.3 [26] and RStudio version 1.3.1093 [27] software. A total of 8.807 participants presenting complete data for all variables of interest in the study were considered for analysis. To reduce the differences in estimates that would arise with the exclusion of observations, the complete cases in the database were weighted using the Inverse Probability Weighting (IPW) procedure. In our study, we adopted the IPW due to its robust handling of Missing at Random (MAR) data, a condition aligned with our dataset where the probability of missingness relies on other observed variables. Additionally, our weighted data necessitated a method that could efficiently accommodate these weights, and IPW met this need by directly using these weights to adjust for missing data, thereby optimizing our complex survey data handling [28, 29].

To verify the association between independent variables and hospitalisation, prevalence ratios (PR) with their respective 95% confidence intervals (CIs) were calculated using Poisson regression models with robust variance adjusted for the other independent variables. Multiple linear regression models (i.e., adjusted for the other indicators) were also utilised for readmission outcomes and length of stay. β coefficients and a 95% CI were presented.

### Network analysis

For network analysis, the hospitalisation variable concerning the 19 morbidities was included (binary variables: 0 = no, 1 = yes) stratified by sex (male, female) and age group (50 to 59, 60 to 74, and ≥ 75 years). For the network analysis, only the hospitalisation variable was selected as the outcome, as the network models for the other two outcomes (i.e., readmissions and length of stay) tended towards non-convergence when the sample was stratified by independent variables (i.e., sex and age group).

Ising models were used to estimate the networks [30]. Least absolute shrinkage and selection operator (LASSO) and the extended Bayesian information criteria (EBIC) model selection method with a hyperparameter γ of 0.25 were used to regularise and select the most appropriate model, respectively. Non-parametric bootstraps with 500 resamples were used to assess the estimation method’s accuracy through the construction of a 95% CI for each model’s estimated edges. Bootstrap difference tests were used to compare edge magnitudes (Supplementary Material) [31, 32]. The Walktrap community detection algorithm, available in the igraph package, was utilized to estimate network communities. This algorithm operates on the principle that short random walks are likely to remain within the same community. These short random walks were used to calculate the similarity between nodes, which were grouped based on this characteristic [33]. To check the stability of communities in all groups, the eigenvalues were verified, which provided similar results for all groups. Moreover, in this study, bootstrapping was employed to guarantee the stability of community estimation. The algorithm was executed 100 times on bootstrapped samples with replacement. Subsequently, the median value of the community samples was determined, and the nodes membership was randomly selected. Community analysis provides a holistic view of multimorbidity by evaluating co-occurring diseases and revealing potential shared risk factors or mechanisms.

The predictability measure correct classification (CC) was computed for each network node, and the centrality measures strength, closeness, betweenness, and participation coefficient were calculated [34, 35]. Case-dropping subset bootstraps with 500 resamples, correlation stability coefficients (CS-coefficients), and bootstrap difference tests were performed to respectively assess the stability of the centrality measures and compare them (Supplementary Material) [32].

Regarding network visualisation and interpretation, edge thickness indicates the connection between the nodes. The size of the nodes varies according to the prevalence of the variables. The flow algorithm was applied (when possible) to position the nodes in the networks, with the hospitalisation variable defined as the target node in all figures.

## Results

When we analysed the association between multimorbidity ≥ 2 and hospitalisation we found the risk factors in males PR = 1,26 (1,02–1,55), aged ≥ 75 years PR = 1,35 (1.08, 1.69**)** and in females aged ≥ 75 years PR = 1,42 (1.07, 1.89). Regarding multimorbidity ≥ 2 and hospitalisation the association was only found in males PR = 1,29 (1.05, 1.58) (Table 1).

Regarding the association of readmission with multimorbidity (MM2 and MM3) there are no statistical significance in the stratification by sex and age. However, for length of stay under both definitions of multimorbidity (MM2 and MM3) statistical significance were observed in males (**β** > 3) and females aged ≥ 75 (**β** > 2,5), all betas with respective 95% IC as a risk factors for this outcome (Table 2).

**Table 1:** Prevalence of hospitalisation and its association with sex, age group according to the presence of multimorbidity (≥ 2 or ≥ 3 chronic conditions). The Brazilian Longitudinal Study of Ageing (ELSI-Brazil), 2015–2016. (n = 8807)

Multimorbidity ≥ 2 (n = 6088)				
Independent variables	*n*	% (95%CI)	Hospitalisation (n = 725)	
			% (95%CI)	PR (95%CI)
**Sex**				
Female	3801	59,8 (57.0, 62.5)	11 (9.7, 12.2)	1,00
Male	2287	40,2 (37,5; 43)	13,7 (11,3; 16)	1,26 (1,02; 1,55)*
**Age group**				
50–59	2224	41,9 (38.0, 45.9)	10,9 (9.4, 12.5)	1,00
60–74	2719	41,9 (39.4, 44.4)	12,2 (10.3, 14.1)	1,11 (0.92, 1.35)
≥ 75	1145	16,2 (14.1, 18.6)	14,6 (12.0, 17.2)	1,35 (1.08, 1.69)*
**Age group - Female**				
50–59	1306	41,4 (37.8, 45.0)	10,3 (8.3, 12.3)	1,00
60–74	1758	41,5 (39.0, 44.0)	10,2 (8.5, 11.8)	0,99 (0.78, 1.26)
≥ 75	737	17,1 (14.8, 19.7)	14,6 (11.5, 17.7)	1,42 (1.07, 1.89)*
**Age group - Male**				
50–59	918	42,7 (37.3, 48.3)	11,9 (9.1, 14.7)	1,00
60–74	961	42,4 (38,6; 46,3)	15,1 (11.3, 19.0)	1,27 (0,95; 1,69)
≥ 75	408	14,9 (12,5; 17,7)	14,7 (9.8, 19.6)	1,23 (0.82, 1.86)
**Multimorbidity ≥ 3 (n = 4299)**				
**Independent variables**	***n***	**% (95%CI)**	**Hospitalisation (n = 572)**	
			**% (95%CI)**	**PR (95%CI)**
**Sex**				
Female	2873	64,4 (61.6, 67.2)	12,2 (10.8, 13.6)	1,00
Male	1426	35,6 (32.8, 38.4)	15,8 (13.0, 18.5)	1,29 (1.05, 1.58)*
**Age group**				
50–59	1387	38 (34.5, 41.6)	12,7 (10,7; 14,7)	1,00
60–74	1978	42,9 (40.7, 45.1)	13,7 (11.4, 16.0)	1,07 (0.86, 1.34)
≥ 75	934	19,1 (16.7, 21.8)	14,7 (12.0, 17.4)	1,16 (0.91, 1.47)
**Age group – Female**				
50–59	897	38,3 (35.1, 41.5)	11,8 (9.4, 14.3)	1,00
60–74	1349	42,2 (39.8, 44.6)	11,5 (9.5, 13.4)	0,97 (0.74, 1.28)
≥ 75	627	19,5 (16.9, 22.5)	14,6 (11.3, 17.9)	1,24 (0.9, 1.7)
**Age group – Male**				
50–59	490	37,5 (32.2, 43.2)	14,2 (10.8, 17.7)	1,00
60–74	629	44 (40.3, 47.8)	17,5 (12.7, 22.4)	1,23 (0.89, 1.7)
≥ 75	307	18,5 (15.5, 21.8)	14,8 (9.7, 19.8)	1,04 (0.68, 1.58)

*Notes:* 95%CI: 95% confidence interval. PR: Prevalence ratio. n: Unweighted number of choices. Relative frequencies (%) and weighted confidence intervals. * p < 0.05

**Table 2:** Readmission and length of stay of hospitalised individuals and associations adjusted with the variables sex, age group according to the presence of multimorbidity assessed as ≥ 2 or ≥ 3 chronic conditions. The Brazilian Longitudinal Study of Ageing (ELSI-Brazil), 2015–2016. (n = 887)

Multimorbidity ≥ 2 (n = 725)						
Independent variables	*n*	% (95%CI)	Readmission		Length of stay	
			M (*SD*)	β (95%CI)	M (*SD)*	β (95%CI)
**Sex**						
Female	429	54,3 (49.0, 59.6)	1.6 (1.1)	1,00	4.9 (9.1)	1,00
Male	296	45,7 (40.4, 51.0)	1.6 (1.3)	0,04 (-0.27, 0.34)	8.4 (11.0)	3,56 (1.71, 5.42)*
**Age group**						
50–59	245	38 (31.8, 44.6)	1.6 (1.2)	1,00	6.7 (10.9)	1,00
60–74	310	42,3 (37.2, 47.6)	1.6 (1.4)	-0,02 (-0.36, 0.33)	6.1 (10.6)	-0,73 (-2.81, 1.35)
≥ 75	170	19,7 (15.9, 24.0)	1.4 (0.8)	-0,19 (-0.42, 0.04)	6.8 (7.1)	0,37 (-1.46, 2.19)
**Age group – Female**						
50–59	140	38,7 (31.6, 46.4)	1.7 (1.3)	1,00	3.6 (4.9)	1,00
60–74	180	38,5 (33.0, 44.3)	1.5 (1.2)	-0,21 (-0.57, 0.15)	5.2 (12.5)	1,61 (-0.26, 3.47)
≥ 75	109	22,8 (17.9, 28.5)	1.4 (0.8)	-0,27 (-0.58, 0.03)	6.3 (7.3)	2,69 (1.1, 4.28)*
**Age group – Male**						
50–59	105	37,2 (29.4, 45.6)	1.5 (1.1)	1,00	10.4 (14.5)	1,00
60–74	130	46,8 (38.1, 55.8)	1.7 (1.6)	0,19 (-0.39, 0.78)	7.0 (8.3)	-3,39 (-7.03, 0.24)
≥ 75	61	16 (10.7, 23.1)	1.4 (0.8)	-0,1 (-0.43, 0.24)	7.5 (6.7)	-2,92 (-6.65, 0.82)
**Multimorbidity ≥ 3 (n = 572)**						
**Independent variables**	***n***	**% (95%CI)**	**Readmission**		**Length of stay**	
			**M (** ***SD*** **)**	**β (95%CI)**	**M (** ***SD*** **)**	**β (95%CI)**
**Sex**						
Female	357	58,4 (53.3, 63.3)	1.6 (1.2)	1,00	4.9 (9.5)	1,00
Male	215	41,6 (36.7, 46.7)	1.7 (1.5)	0,11 (-0.29, 0.51)	7.9 (8.8)	3,1 (1.61, 4.6)*
**Age group**						
50–59	178	35,7 (29.2, 42.7)	1.7 (1.3)	1,00	5.8 (7.7)	1,00
60–74	254	43,5 (37.9, 49.2)	1.7 (1.5)	-0,01 (-0.44, 0.42)	6.1 (11.2)	0,03 (-1.79, 1.84)
≥ 75	140	20,8 (17.1, 25.2)	1.5 (0.8)	-0,24 (-0.54, 0.06)	6.7 (7.5)	1,04 (-0.61, 2.69)
**Age group – Female**						
50–59	109	37 (29.7, 45.0)	1.8 (1.3)	1,00	3.7 (5.2)	1,00
60–74	155	39,6 (33.8, 45.8)	1.6 (1.2)	-0,2 (-0.66, 0.26)	5.1 (13.0)	1,37 (-0.61, 3.35)
≥ 75	93	23,4 (18.5, 29.0)	1.5 (0.8)	-0,28 (-0.69, 0.13)	6.3 (7.6)	2,54 (0.88, 4.2)*
**Age group – Male**						
50–59	69	33,8 (25.4, 43.4)	1.7 (1.2)	1,00	9.1 (9.5)	1,00
60–74	99	48,9 (38.8, 59.0)	1.9 (1.8)	0,25 (-0.52, 1.02)	7.2 (8.7)	-1,86 (-4.82, 1.11)
≥ 75	47	17,3 (11.5, 25.2)	1.5 (0.9)	-0,19 (-0.64, 0.25)	7.6 (7.3)	-1,49 (-4.56, 1.58)

*Notes:* 95%CI: 95% confidence interval. PR: Prevalence ratio. n: Unweighted number of choices. Relative frequencies (%) and weighted confidence intervals. M: mean. SD: standard deviation * p < 0,05

**Fig. 1:**
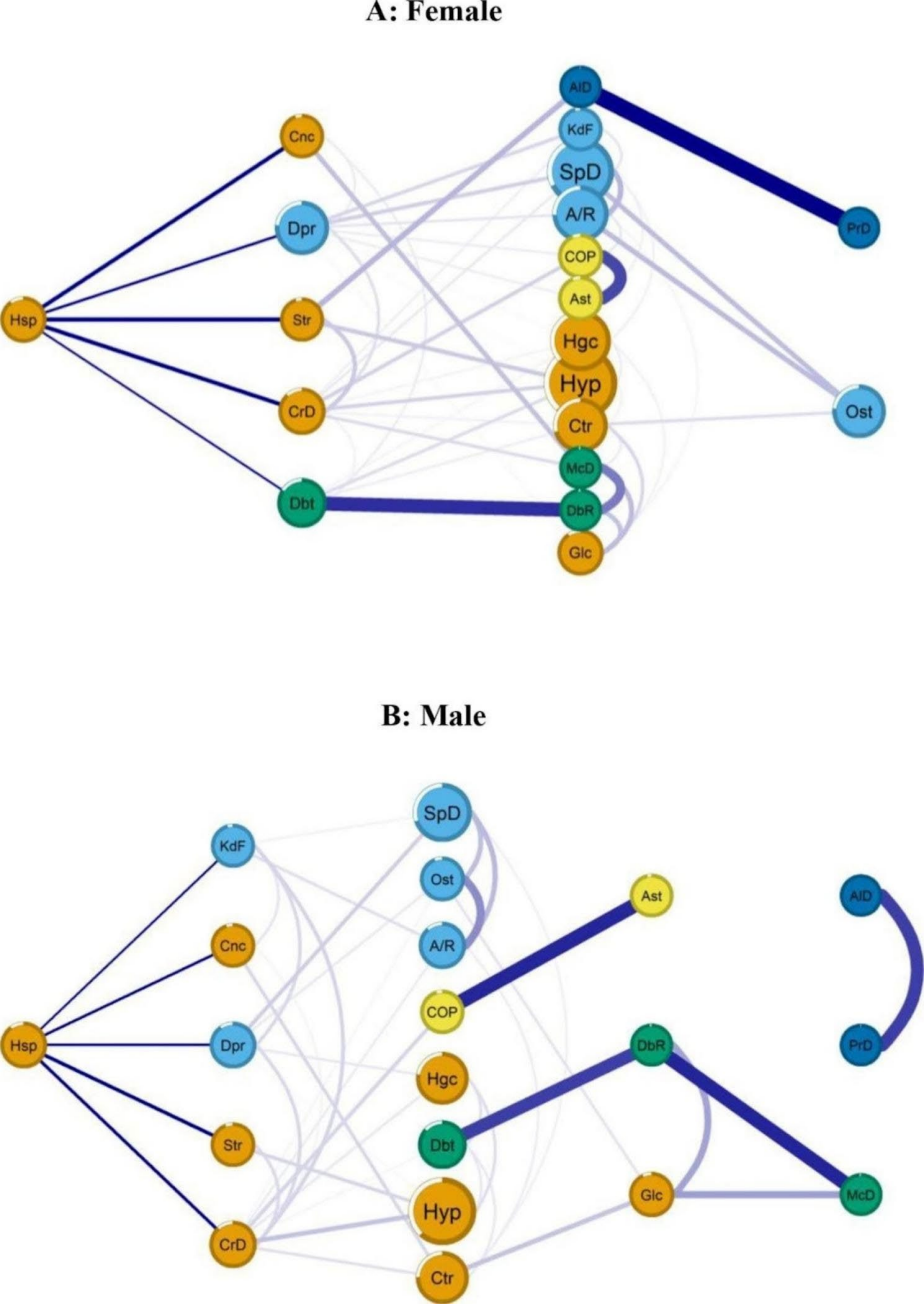
Flow networks of morbidities and hospitalisation variables stratified by sex. The Brazilian longitudinal study of ageing (ELSI-Brazil), 2015 – 2016

In the networks of connections between morbidities and hospitalisation among females (*n* = 4.960) and males (*n* = 3.847), we observed higher frequencies of hypertension (female: 55.7%; male: 47.7%) and back problems (female: 46.2%; male: 33.5%) (Fig. 1 and Supplemental Table S1). Community analysis identified five groups of diseases: Group 1 cardiovascular diseases–cancer–cataract–glaucoma (orange), Group 2 musculoskeletal diseases–depression–kidney failure (light blue), Group 3 diabetes and related complications (green), Group 4 respiratory diseases (yellow), and Group 5 neurodegenerative diseases (Alzheimer’s and Parkinson’s) (dark blue) (Fig. 1). The Group 1 showed strong connections with the outcome of hospitalisation.

In the hospitalisation and multimorbidity networks stratified by sex, we observed three layers of variables directly or indirectly connected to hospitalisation. For the females, the variables directly connected to the outcome variable were cancer, depression, stroke, heart disease, and diabetes. For males, the variables directly connected to hospitalisation were kidney failure, cancer, depression, stroke, and heart disease. Among females, we observed mediating variables between hospitalisation and other morbidities; that is, stroke mediated Alzheimer’s disease, depression mediated renal failure, heart disease mediated hypertension, and diabetic retinopathy mediated diabetes in connection with hospitalisation. Among males, depression mediated between back problems and hospitalisation (Fig. 1).

**Fig. 2:**
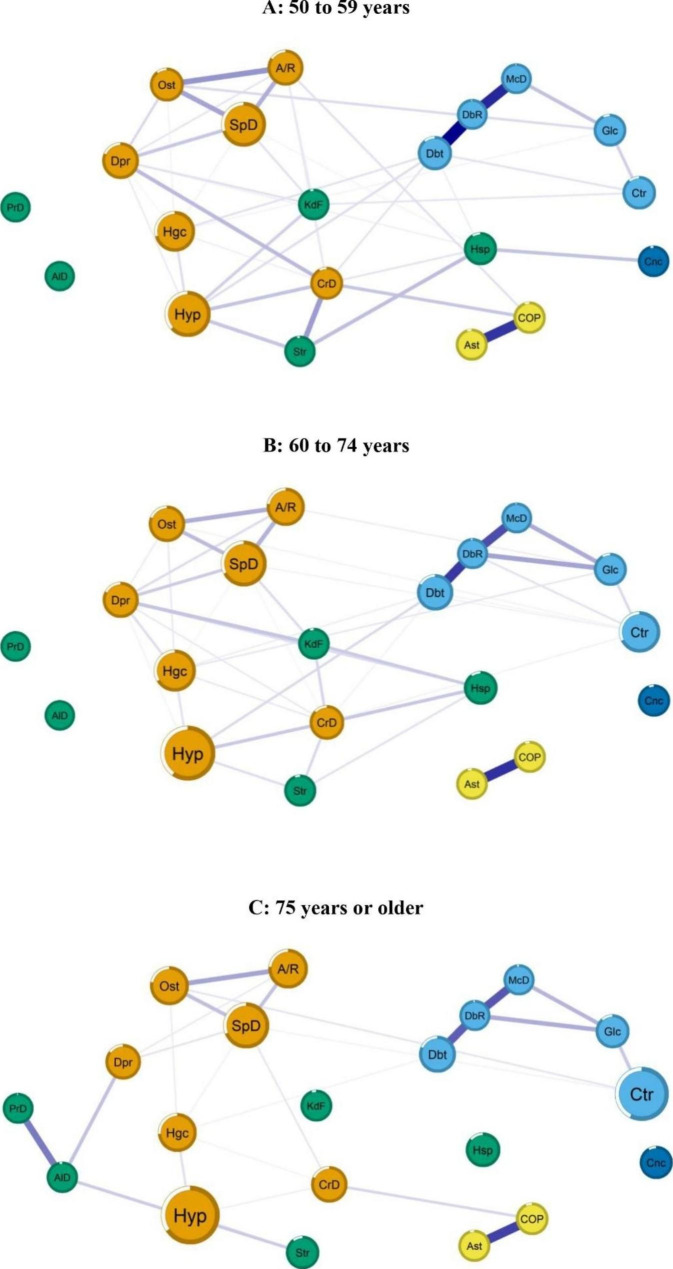
Networks of morbidities and hospitalisation variables stratified by age. The Brazilian longitudinal study of ageing (ELSI-Brazil), 2015 – 2016

The most prevalent morbidities in the networks stratified by age group were as follows: Among persons aged 50 to 59 years (*n* = 3.778), 41.9% had hypertension and 39.0% had back problems; among persons aged 60 to 74 years (*n* = 3.624), 59.6% had hypertension and 41.3% had back problems; and among persons aged 75 years and older (*n* = 1.405), 66.3% had hypertension and 57.8% had a cataract (Supplemental Table S2). Community analysis by age group identified five disease groups: Group 1 cardiovascular–musculoskeletal diseases–depression (orange), Group 2 diabetes and related complications (light blue), Group 3 neurodegenerative diseases–renal failure–haemorrhagic stroke (green), Group 4 respiratory diseases (yellow), and Group 5 cancer (dark blue) (Fig. 2 and Supplemental Table S2). The neurodegenerative diseases–renal failure–haemorrhagic stroke group was the most strongly associated with hospitalisation.

The 50–59 age group’s network was denser (i.e., more connections between nodes) than the other networks. Hospitalisation was mainly connected with the variables cancer, stroke, and heart disease. In the 60–74 age group’s network, hospitalisation was associated with kidney failure, depression, heart disease, and stroke. It was also noted that heart disease and stroke mediated the connection between hypertension and hospitalisation in both networks (Fig. 2).

In the over 75 age group’s network, we did not observe connections between morbidities and hospitalisation. However, it is noteworthy that Parkinson’s disease and Alzheimer’s disease showed a strong relationship. The latter seems to play a mediating role between the hypertension and depression variables.

## Discussion

In this study, we highlighted that there is influence of sex and age modifying the association between multimorbidity and hospitalisation variables - occurrence, readmission, and length of stay. The associations between MM nodes with the hospitalization node, as well as the positions of the nodes in network structures, changed with stratification by sex and age group.Length to stay also had association in subgroup analyses by age and sex, with highest medium of stay in males and females with ≥ 75 years old. Thus, we emphasise the importance of stratification by sex and age group in studies on multimorbidity and hospitalisation among adults and older adults aged ≥ 50 years to better understand the changes in this association within multimorbidity networks according to these variables.

This study verified the association between male sex and hospitalisation under both definitions of multimorbidity. However, concerning the female sex, this association was found only in the ≥ 75 age group and with MM2. Hospitalisation among those with MM2 was associated with being aged ≥ 75, regardless of sex. These results corroborate those of other studies showing that sociodemographic factors such as sex and age may influence the risk of hospitalisation among persons with multimorbidity [5, 6]. However, we could not locate detailed studies such as ours that would allow us to deepen these comparisons.

Regarding evidence of the association between multimorbidity and hospitalisation in men, preventive actions focusing on men are necessary. In Brazil, since 2009, the National Policy for Integral Attention to Men’s Health was implemented with a view to driving actions to promote health within this population, specifically to reduce their higher rates of morbidity and mortality [36]. Given that men have difficulty seeking preventive health services [37], these public policies are important to reduce male hospitalisation rates.

The association between multimorbidity (MM2 and MM3) and readmission and length of stay was demonstrated again in men and women aged ≥ 75 years. In line with the literature, multimorbidity increases the risk of hospital readmissions as well as the length of stay among older adults [10, 11, 38]; however, to our knowledge, few studies have investigated these associations according to sex. A systematic review of factors associated with hospital readmission in older adults did not identify a significant association with sex and age [39]. These results reinforce the importance of individualised care, taking each sex’s specific aspects into account and identifying the influence of sex and age in the association between multimorbidity, and length of stay.

Network analysis showed that hypertension and back problems were the most prevalent morbidities in the studied sample and that among those aged ≥ 75 years, cataracts were more prevalent. A cross-sectional study of nine countries’ ≥ 50 populations also found a high prevalence of hypertension (> 59.0%, except in India, which was 37.51%) and cataracts, except for South Africa [40]. A study involving the general population of six Latin American and Caribbean countries (Brazil, Colombia, El Salvador, Jamaica, Mexico, and Panama) found that 32.3% of hypertensive individuals had at least one additional chronic condition [41]. In southern Brazil, the most prevalent morbidity dyad in the older adult population was hypertension and back problems (23.6%), a finding that is similar to ours. Two systematic reviews of studies on spine problems among the older adult population, ≥ 60 years, revealed a prevalence of more than 20% for this condition [44, 43]. Increasing life expectancy also increases the number of people with age-related eye diseases, with cataracts being the world’s leading cause of visual impairment and blindness [44–46]. Given that a chronic condition can be a risk factor for other conditions as well as for an unfavourable prognosis for care, identifying the most prevalent diseases is essential to improve health actions regarding prevention and/or treatment.

Through stratification by age group, we found a connection between hospitalisation and the neurodegenerative diseases–renal failure–haemorrhagic stroke disease group. In Spain, a study on sex and age differences in multimorbidity prevalence among older adults identified a pattern related to advancing age, which differs from our findings regarding several additional morbidities and the ‘psychogeriatric’ pattern (behavioural problems, cardiac arrhythmia, cerebrovascular disease, chronic skin ulcer, congestive heart failure, dementia and delirium, iron deficiency, osteoporosis, Parkinson’s disease) [47]. More attention should be paid to age-related diseases to mitigate their effects on health, especially among individuals affected by neurodegenerative diseases, renal failure, and haemorrhagic stroke who have a high morbidity burden.

We identified five groups of diseases associated with hospitalisation when stratified by sex, highlighting the cardiovascular–cancer–cataract–glaucoma group. Similar to a Spanish study conducted among older adults aged ≥ 64 from primary care centres in two southern European regions of Spain (Aragon and Catalonia), the cardiometabolic disease group (atherosclerosis, cardiac arrhythmia, congestive heart failure, diabetic gout, haematological disorders, hypertension, ischemic heart disease, iron deficiency, obesity, and other cardiovascular disorders) was more prevalent across both sexes. However, this study did not analyse the multimorbidity pattern associated with hospitalisation [47]. Cardiovascular diseases are risk factors for other conditions such as cataracts, glaucoma, and cancer [48, 49]. Thus, the pattern observed in our study is consistent. Regarding cancer, the treatment itself may cause cardiovascular toxicity, generating cardiac problems [50]. In addition to this study’s finding of an association of cardiovascular diseases with hospitalisation, cardiovascular disease is the leading cause of death in Brazil and worldwide. Thus, primary health care once again proves essential for health promotion and prevention of the incidence of risk factors for cardiovascular diseases [51] among adults and older adults aged ≥ 50 years.

Although the cardiovascular–cancer–cataract–glaucoma disease group had strong connections with hospitalisation across both sexes, these diseases’ interaction patterns differed by sex. In females, the diseases directly connected to hospitalisation were cancer, depression, stroke, heart disease, and diabetes, while in males; they were kidney failure, cancer, depression, stroke, and heart disease. Thus, we verified sex differences regarding two diseases: diabetes in females and renal failure in males. In a retrospective cohort study conducted among older adult patients aged ≥ 75 years hospitalised at University Hospital Mutua de Terrassa (Spain), chronic kidney disease was also more prevalent among hospitalised men (47%). However, diabetes had a similar prevalence (27.5% in males and 26.6% in females). In the same study, the cardiovascular disease group was the second most prevalent across both sexes, and the metabolic disease group was the most prevalent among hospitalised older adults [52]. By identifying the differences in the multimorbidity pattern associated with hospitalisation, we can better shape health strategies to prevent hospitalisation among older adults and mitigate their health consequences.

Node centrality measures were used to examine the structural significance of each disease in the network to determine which diseases would be the most effective intervention targets, i.e., those that would have the greatest impact on the model. Our findings show that the direct effect of stroke and Alzheimer’s on the structure of the morbidities network varies by gender, with a stronger influence on women’s networks. According to the model, these two morbidities remain the most probable most effective point for action to avoid hospitalization in women. Understanding this information provided by network centrality measurements can make a difference in clinical management for these individuals preventing hospitalization.

This study is limited by its lack of temporality, making it impossible to establish causality in the assessed associations, which is intrinsic to cross-sectional studies. Additionally, using self-reported information for 19 morbidities may lead to discussions about information bias, however it is commonly used in epidemiological studies to analyse multimorbidity. Network analysis helps clarify several aspects, but it is impossible to identify which diseases specifically lead to the emergence of others. Therefore, this type of analysis of this study allows us to better visualise the patterns of multimorbidity that are associated with hospitalisation, facilitating an understanding of these interrelations. By identifying these patterns, it becomes possible to improve and direct health strategies and actions related to the care of people with multimorbidity. We emphasise that the ELSI-Brazil study relied upon a representative sample of the Brazilian population aged 50 years or older, increasing the reliability of these results and their external validity.

We recommend that future research verify which diseases contribute to the emergence of others and which of them are associated with a higher risk of hospitalisation. Identifying patterns of multimorbidity associated with hospitalisation is relevant for local health care planning, prioritisation of health care and resources, development of public policies, and improvement of health actions in primary care. We emphasise the importance of stratifying this association by age and sex to identify the existing differences and thus provide personalised care to different populations.

## Conclusion

We concluded that the association between hospitalisation, readmission, length of stay, and multimorbidity differs according to sex and age group. Male sex increased hospitalisation risk, readmission, and length of stay under both definitions of multimorbidity (MM2 and MM3). Hospitalisation risk, readmission, and length of stay were all higher in the 75 and older age group, both overall and among women. Regarding the multimorbidity pattern, the neurodegenerative diseases–renal insufficiency–haemorrhagic stroke group was strongly associated with hospitalisation when stratified by age group, as was the cardiovascular diseases–cancer–cataract–glaucoma group when stratified by sex.

### Electronic supplementary material

Below is the link to the electronic supplementary material.

Supplementary Material 1

Supplementary Material 2

Supplementary Material 3

Supplementary Material 4

Supplementary Material 5

Supplementary Material 6

## Data Availability

The data is available on the website: <http://elsi.cpqrr.fiocruz.br/>.
