# Supplementary figures and images for: Associations of hospitalisation – admission, readmission and length to stay – with multimorbidity patterns by age and sex in adults and older adults: the ELSI-Brazil study
Source: BMC Geriatr. 2023 Aug 21;23:504. doi: 10.1186/s12877-023-04167-8 (PMC10441711; doi:10.1186/s12877-023-04167-8)

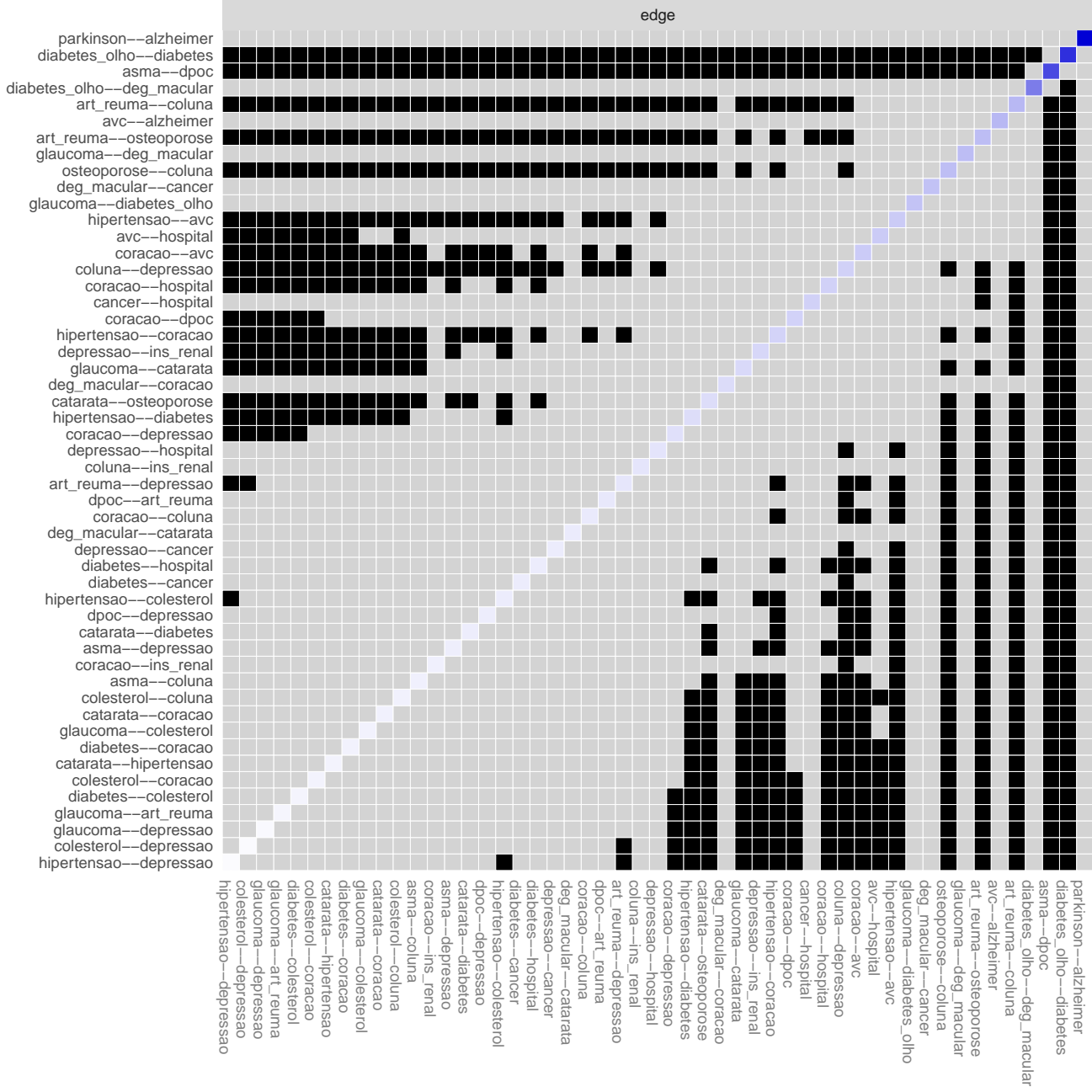

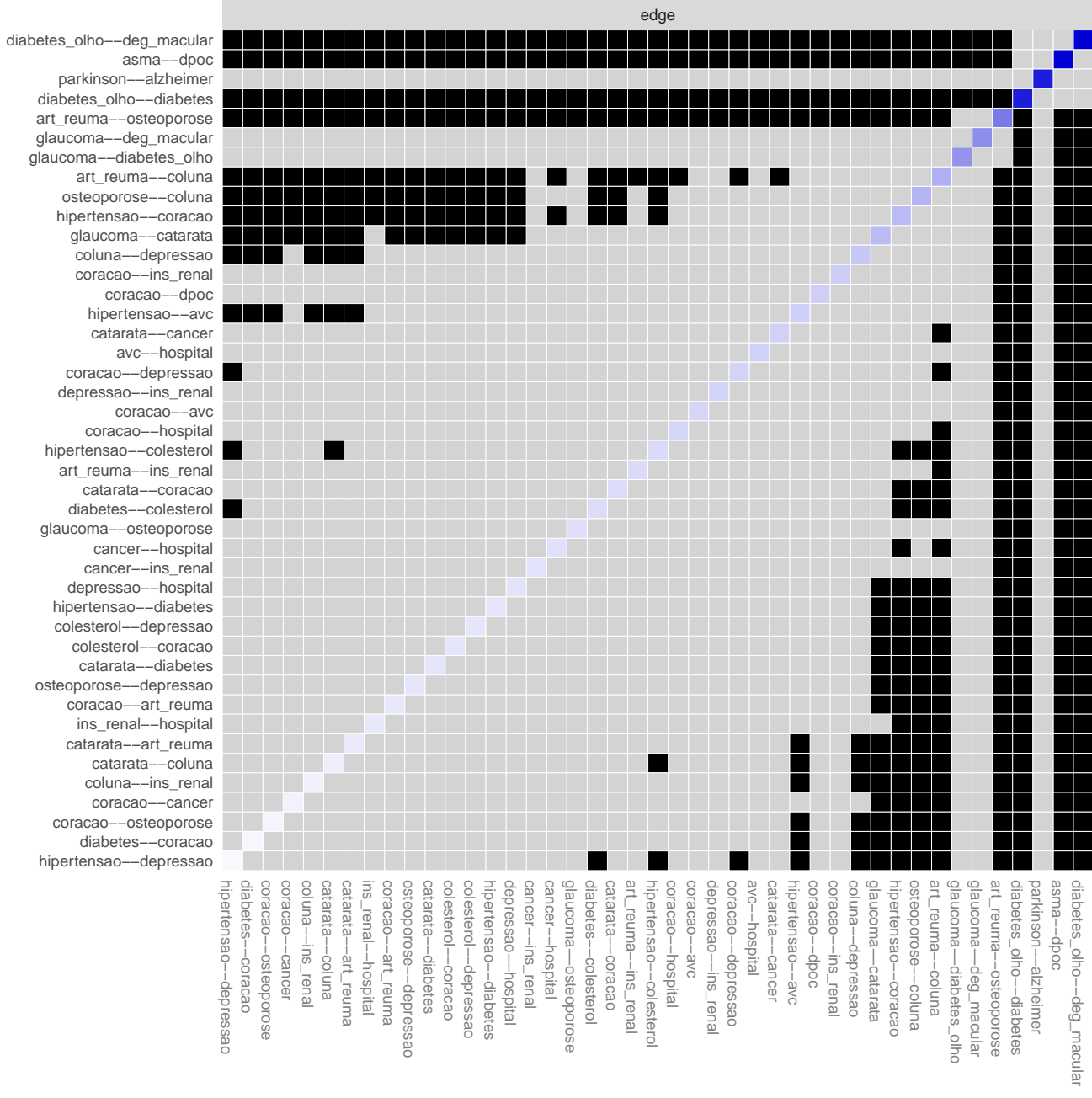

Supplement: Supplementary file 1 — Supplementary Material 1 [file 12877_2023_4167_MOESM1_ESM.pdf]

● Bootstrap mean    ● Sample

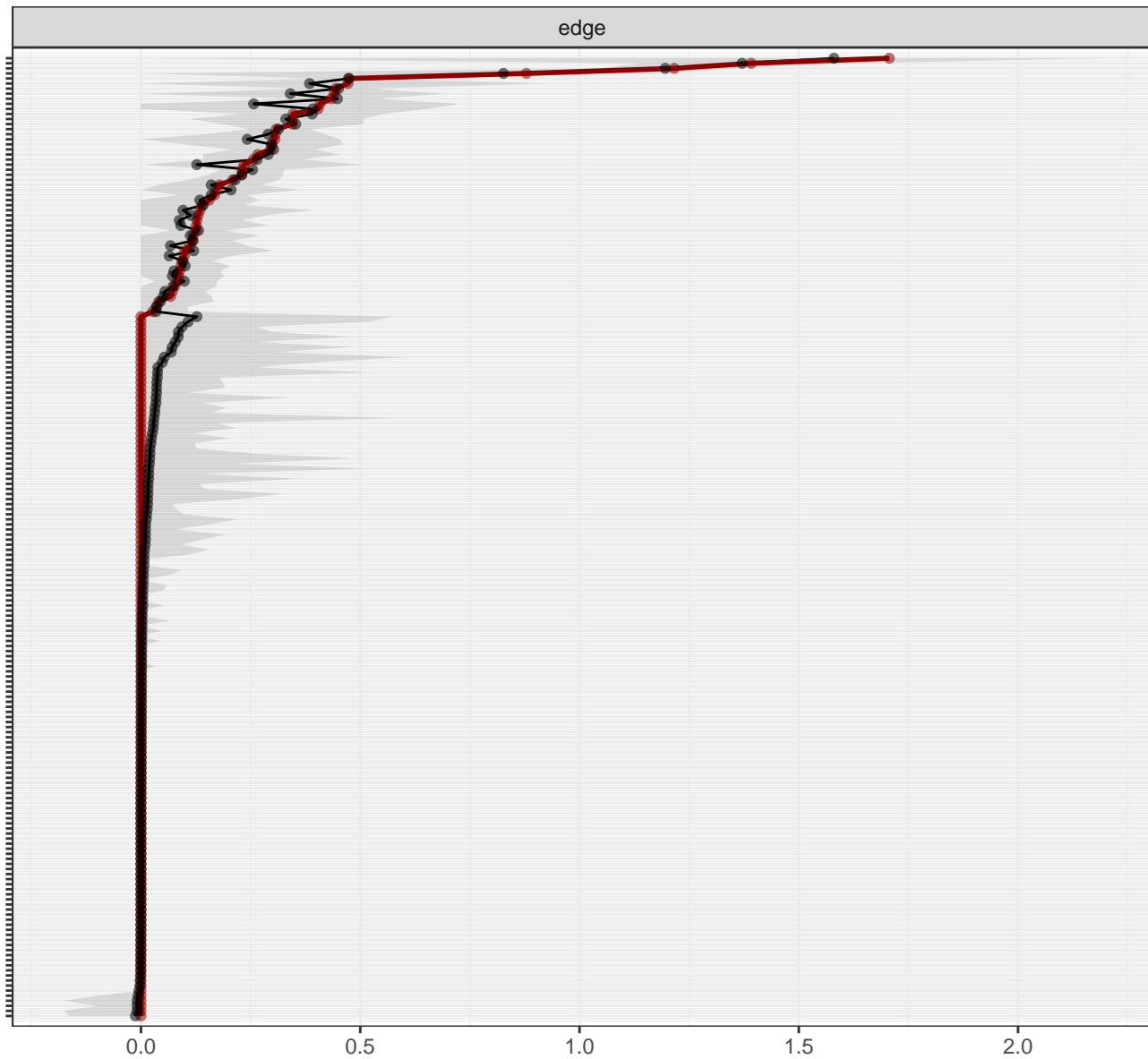

● Bootstrap mean    ● Sample

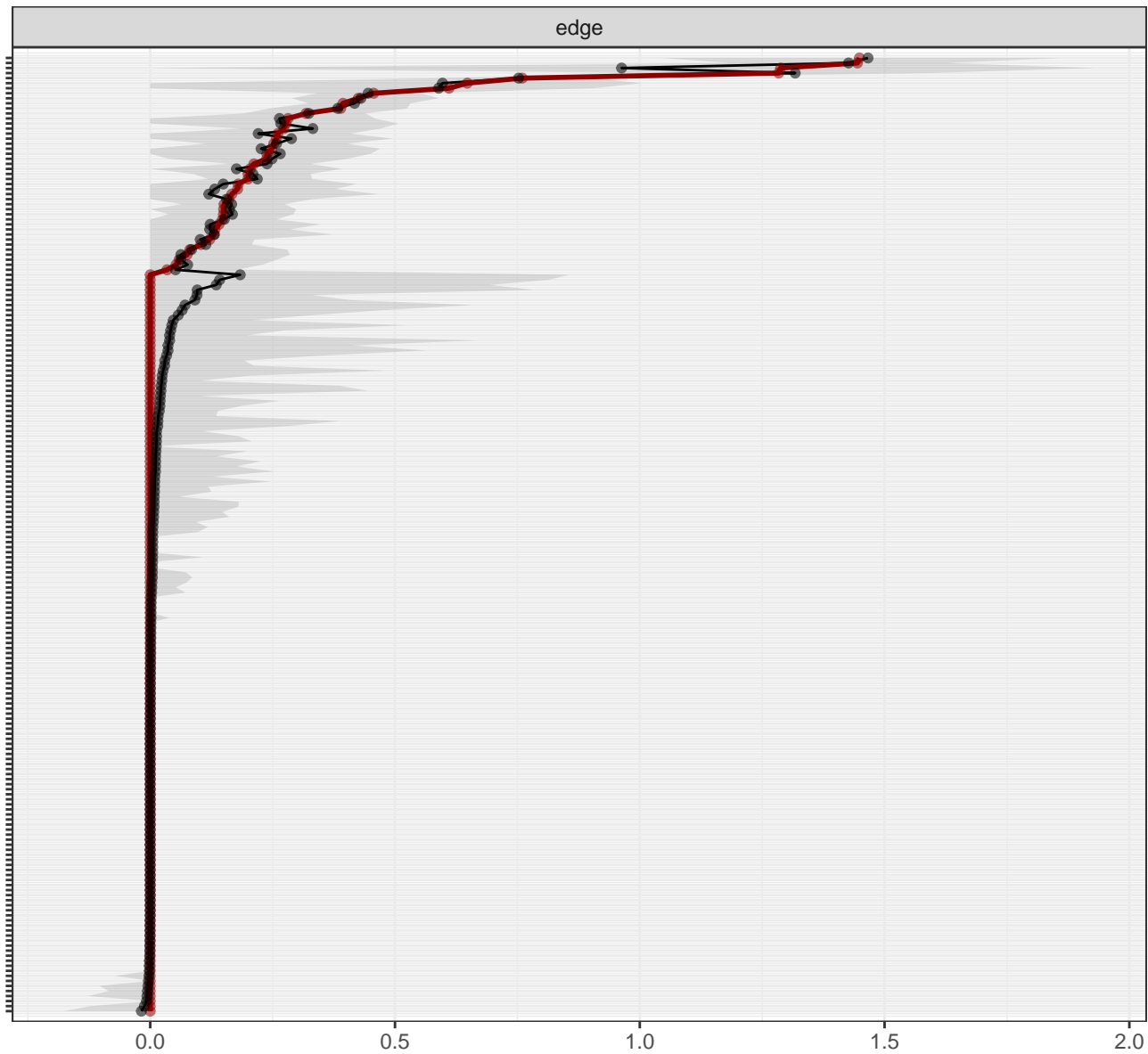

Supplement: Supplementary file 2 — Supplementary Material 2 [file 12877_2023_4167_MOESM2_ESM.pdf]
