# Supplementary material for: Associations of hospitalisation – admission, readmission and length to stay – with multimorbidity patterns by age and sex in adults and older adults: the ELSI-Brazil study
Source: BMC Geriatr. 2023 Aug 21;23:504. doi: 10.1186/s12877-023-04167-8 (PMC10441711; doi:10.1186/s12877-023-04167-8)
Supplement: Supplementary file 3 — Supplementary Material 3 [file 12877_2023_4167_MOESM3_ESM.pdf]

Figure S1. Measures of morbidity centrality and hospitalisation variables stratified by sex. The Brazilian Longitudinal Study of Ageing (ELSI-Brazil), 2015 - 2016.

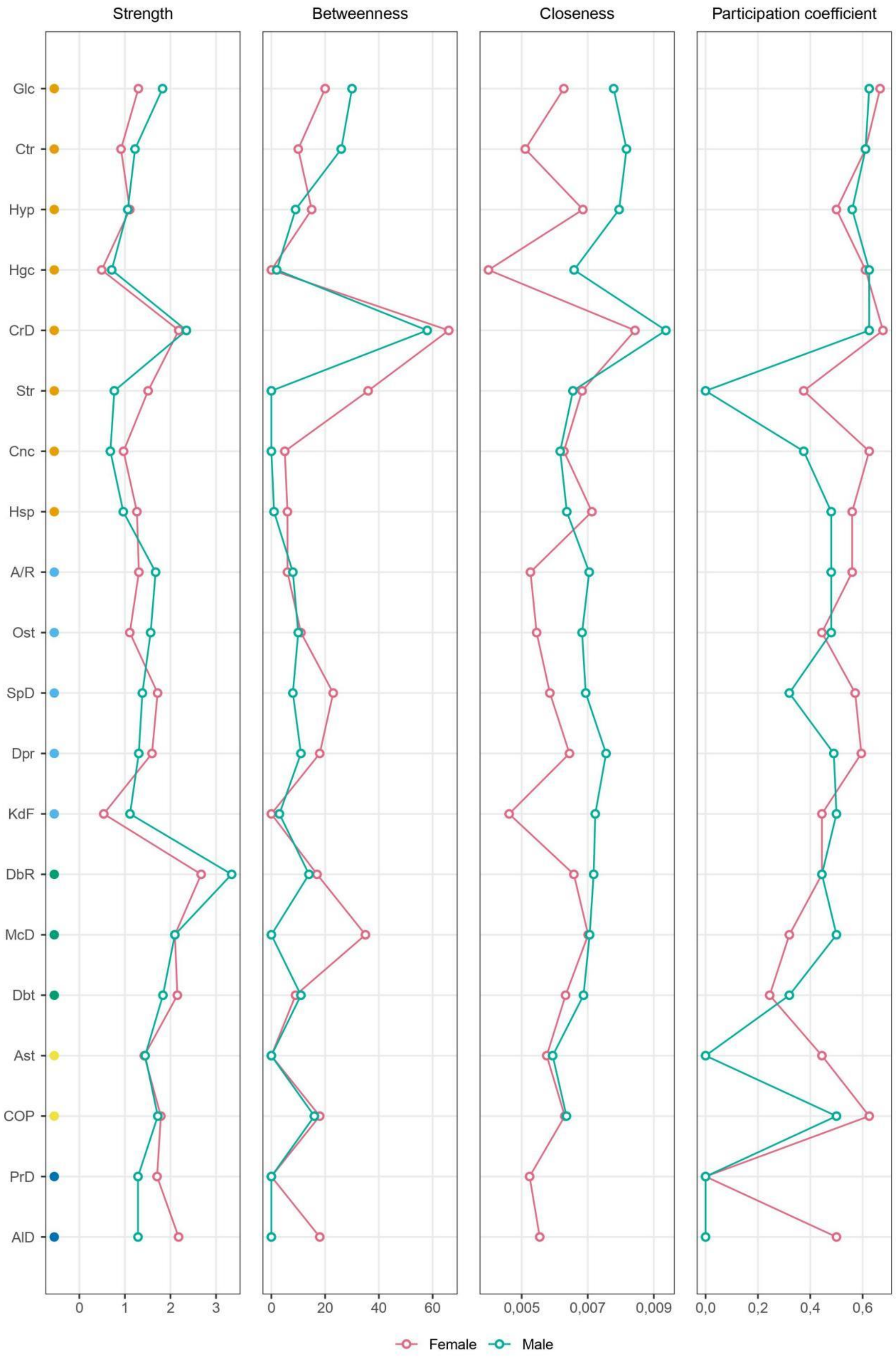

Notes. Disease groups: *Cardiovascular diseases–cancer–cataract–glaucoma* (orange): Glc (Glaucoma), Ctr (Cataract), Hyp (Hypertension), Hgc (High cholesterol), CrD (Heart Disease), Str (Stroke), Cnc (Cancer), Hsp (Hospitalization); *Musculoskeletal diseases–depression–kidney failure (light blue):* A/R (Arthritis / Rheumatism), Ost (Osteoporosis), SpD (Spine Problem), Dpr (Depression), KdF (Kidney Failure); *Diabetes and related complications (green)*: DbR (Diabetic Retinopathy), McD (Macular Degeneration), Dbt (Diabetes); *Respiratory diseases (yellow)*: Ast (Asthma), COP (Chronic obstructive pulmonary disease); *Neurodegenerative diseases (dark blue):* PrD (Parkinson Disease), AlD (Alzheimer Disease).
