# Supplementary material for: Associations of hospitalisation – admission, readmission and length to stay – with multimorbidity patterns by age and sex in adults and older adults: the ELSI-Brazil study
Source: BMC Geriatr. 2023 Aug 21;23:504. doi: 10.1186/s12877-023-04167-8 (PMC10441711; doi:10.1186/s12877-023-04167-8)
Supplement: Supplementary file 4 — Supplementary Material 4 [file 12877_2023_4167_MOESM4_ESM.pdf]

Figure S2. Measures of morbidity centrality and hospitalisation variables stratified by age groups. The Brazilian Longitudinal Study of Ageing (ELSI-Brazil), 2015 - 2016.

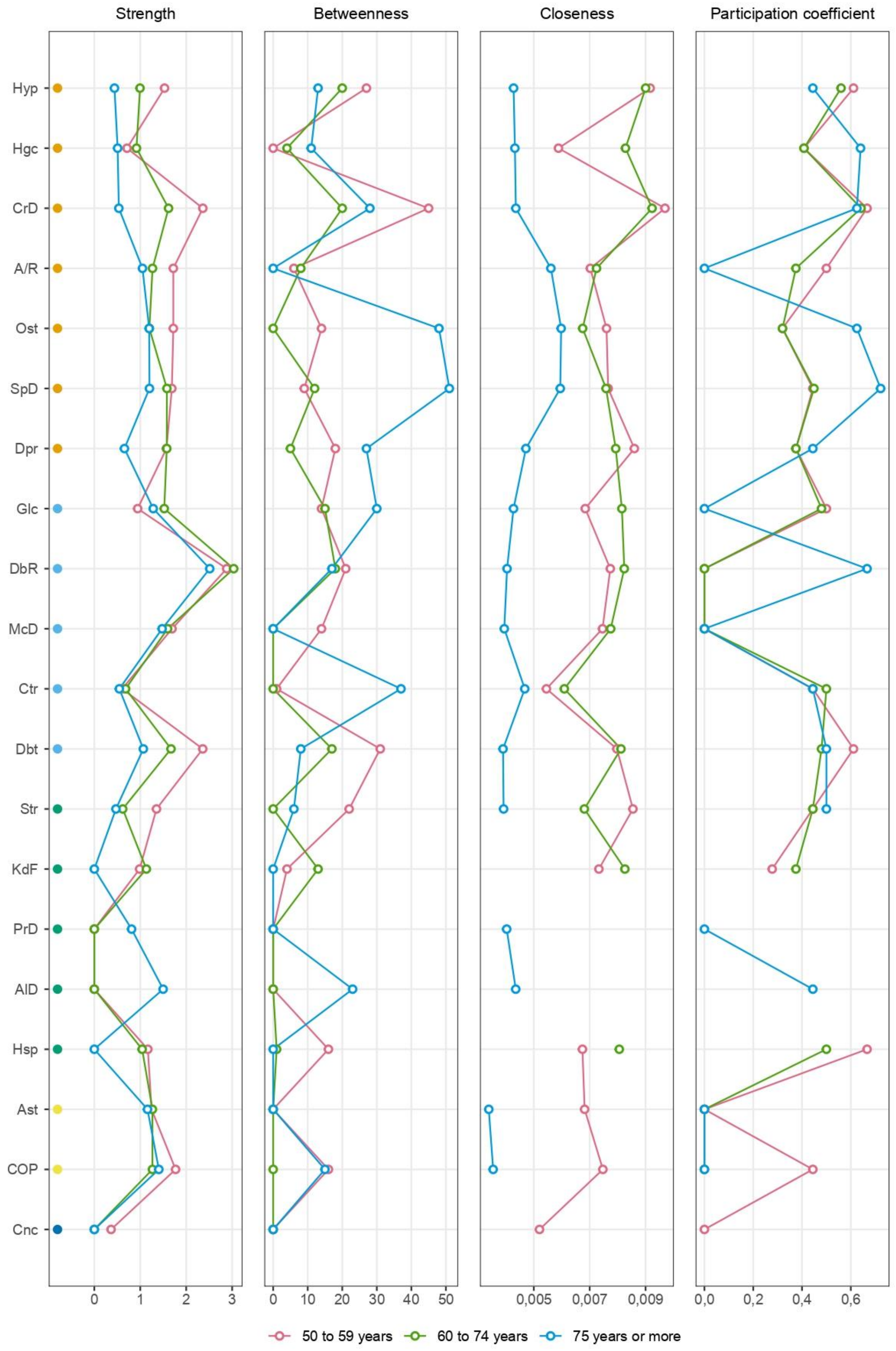

Notes. Disease groups: *Cardiovascular–musculoskeletal diseases–depression (orange)*: Hyp (Hypertension), Hgc (High Cholesterol CrD (Heart Disease), A/R (Arthritis / Rheumatism), Ost (Osteoporosis), SpD (Spine Problem); Dpr (Depression); *Diabetes and related complications (light blue)*: Glc (Glaucoma), DbR (Diabetic Retinopathy), McD (Macular Degeneration), Ctr (Cataract), Dbt (Diabetes); *Neurodegenerative diseases–renal failure–haemorrhagic stroke (green):* Str (Stroke), KdF (Kidney Failure), PrD (Parkinson Disease), AID (Alzheimer Disease), Hsp (Hospitalization); *Respiratory diseases (yellow):* Ast (Asthma), COP (Chronic obstructive pulmonary disease); *Cancer* (dark blue): Cnc (Cancer).
