# Supplementary material for: Associations of hospitalisation – admission, readmission and length to stay – with multimorbidity patterns by age and sex in adults and older adults: the ELSI-Brazil study
Source: BMC Geriatr. 2023 Aug 21;23:504. doi: 10.1186/s12877-023-04167-8 (PMC10441711; doi:10.1186/s12877-023-04167-8)
Supplement: Supplementary file 5 — Supplementary Material 5 [file 12877_2023_4167_MOESM5_ESM.pdf]

Table S1. Prevalence and centrality measure of hospitalization network nodes stratified by sex. The Brazilian Longitudinal Study of Ageing (ELSI-Brazil), 2015 - 2016.

Disease Groups	Colour	Prevalence (%)		Centrality Measure	
		Female	Male	Female	Male
**Cardiovascular diseases–cancer–cataract–glaucoma**					
Hyp – Hypertension	● (orange)	55.7	47.7	0.61	0.62
Hgc – High cholesterol	● (orange)	36.7	22.3	0.63	0.77
Ctr – Cataract	● (orange)	25.9	22.7	0.71	0.75
CrD – Heart disease	● (orange)	11.3	11.5	0.88	0.88
Hsp - Hospitalization	● (orange)	9.5	10.4	0.9	0.9
Glc - Glaucoma	● (orange)	9.2	6.9	0.9	0.93
Str – Stroke	● (orange)	4.5	5.7	0.95	0.94
Cnc - Cancer	● (orange)	4.9	5.5	0.95	0.95
**Musculoskeletal diseases–depression–kidney failure**					
SpD – Spine problem	● (blue)	46.2	33.5	0.68	0.7
A/R - Arthritis/rheumatism	● (blue)	28.9	11.2	0.74	0.88
Dpr - Depression	● (blue)	24.9	10.2	0.77	0.9
Ost - Osteoporosis	● (blue)	23.8	6.1	0.77	0.94
KdF – Kidney failure	● (blue)	4.4	4.0	0.96	0.96
**Diabetes and related complications**					
Dbt - Diabetes	● (green)	16.4	14.4	0.84	0.87
DbR – Diabetic retinopathy	● (green)	1.7	1.8	0.98	0.98
McD – Macular degeneration	● (green)	1.6	1.4	0.98	0.99
**Respiratory diseases**					

COP – Chronic obstructive pulmonary disease		6.7	4.8	0.94	0.95
Ast – Asthma		6.3	3.3	0.94	0.96
**Neurodegenerative diseases**					
PrD – Parkinson disease		0.5	0.9	1.0	0.99
AID – Alzheimer disease		0.8	0.4	0.99	1.0
