# Supplementary material for: Associations of hospitalisation – admission, readmission and length to stay – with multimorbidity patterns by age and sex in adults and older adults: the ELSI-Brazil study
Source: BMC Geriatr. 2023 Aug 21;23:504. doi: 10.1186/s12877-023-04167-8 (PMC10441711; doi:10.1186/s12877-023-04167-8)
Supplement: Supplementary file 6 — Supplementary Material 6 [file 12877_2023_4167_MOESM6_ESM.pdf]

Tabela S2. Prevalence and centrality measure of hospitalization network nodes stratified by age groups. The Brazilian Longitudinal Study of Ageing (ELSI-Brazil), 2015 - 2016.

Disease Groups	Colour	Prevalence (%)			Centrality Measure		
		50-59	60-74	≥ 75	50-59	60-74	≥ 75
**Cardiovascular–musculoskeletal diseases–depression**							
Hyp – Hypertension		41.9	59.6	66.3	0.63	0.6	0.66
Hgc – High cholesterol		29.4	32.7	24.7	0.71	0.66	0.74
CrD – Heart disease		8.0	13.2	18.5	0.92	0.87	0.82
A/R - Arthritis/rheumatism		17.1	23.1	26.8	0.84	0.78	0.77
Ost - Osteoporosis		10.9	18.4	24.9	0.89	0.81	0.78
SpD – Spine problem		39.0	41.3	41.9	0.7	0.67	0.69
Dpr - Depression		19.3	17.3	15.9	0.81	0.83	0.85
**Diabetes and related complications**							
Glc - Glaucoma		7.3	8.2	11.3	0.93	0.91	0.88
DbR – Diabetic retinopathy		1.4	2.1	2.1	0.99	0.98	0.98
McD – Macular degeneration		1.3	1.5	2.3	0.99	0.99	0.98
Ctr – Cataract		10.0	30.9	57.8	0.89	0.68	0.57
Dbt - Diabetes		12.2	18.1	19.6	0.89	0.83	0.81
**Neurodegenerative diseases–renal failure– stroke**							
Str – Stroke		3.4	5.5	9.7	0.97	0.94	0.9
KdF – Kidney failure		4.1	4.2	4.8	0.96	0.96	0.95
PrD – Parkinson disease		0.5	0.7	1.7	1.0	0.99	0.98
AID – Alzheimer disease		0.1	0.4	3.2	1.0	1.0	0.97
Hsp - Hospitalization		8.6	10.5	13.3	0.91	0.9	0.86

**Respiratory diseases**							
Ast – Asthma		5.2	4.3	5.4	0.95	0.95	0.95
COP – Chronic obstructive pulmonary disease		6.1	5.0	7.1	0.95	0.95	0.93
**Cancer**							
Cnc - Cancer		3.7	5.9	8.6	0.96	0.94	0.92
